# Are We Anesthesiologists, Aware About the Incidence of Muscle Stiffness Associated With Remifentanil?

**DOI:** 10.5812/kowsar.22287523.3760

**Published:** 2012-01-01

**Authors:** Gauhar Afshan

**Affiliations:** 1 Department of Anaesthesia, Aga Khan University, Karachi, Pakistan

**Keywords:** Propofol, Alfentanil, Remifentanil

Dear Editor, 

I read with interest the article by Imani et al. titled “Use of Remifentanil and Alfentanil in Endotracheal Intubation: A Comparative Study” (1). This prompted me to review existing literature in order to answer whether an induction dose of propofol can reduce the muscle rigidity associated with remifentanil at 5 μg/kg? And what is the minimum dose of remifentanil required for intubation in order to avoid the use of a muscle relaxant? I was amazed that very little literature is available in this regard. Remifentanil is a selective μ-opioid receptor agonist with a very short half-life however, and remifentanil mediated muscle rigidity is a problem that has been well documented in the literature (2). In the article by Imani et al. they did not report any muscle rigidity or difficult ventilation, etc. when propofol was used with remifentanil in fact they found much better intubating conditions with remifentanil when compared to alfentanil. They suggested that remifentanil is the most appropriate opioid for intubating conditions if it is to be used with propofol (1). After reading this article we can see its indication in many day care procedures especially for patients with a history of myopathy or cases in which muscle relaxants are contraindicated. However it is important to be aware that remifentanil should be administered with an agent like propofol as only the combination can avoid the fatal side effect of remifentanil, i.e. muscle rigidity.
